# Targeting c-KIT (CD117) by dasatinib and radotinib promotes acute myeloid leukemia cell death

**DOI:** 10.1038/s41598-017-15492-5

**Published:** 2017-11-10

**Authors:** Sook-Kyoung Heo, Eui-Kyu Noh, Jeong Yi Kim, Yoo Kyung Jeong, Jae-Cheol Jo, Yunsuk Choi, SuJin Koh, Jin Ho Baek, Young Joo Min, Hawk Kim

**Affiliations:** 1 Biomedical Research Center, Ulsan University Hospital, University of Ulsan College of Medicine, Ulsan, 682-060 Republic of Korea; 2 Department of Hematology and Oncology, Ulsan University Hospital, University of Ulsan College of Medicine, Ulsan, 682-714 Republic of Korea; 3 Division of Hematology, Gachon University Gil Medical Center, Gachon University College of Medicine, 21 Namdong-daero 774beon-gil, Incheon, 21565 Republic of Korea

**Keywords:** Drug development, Acute myeloid leukaemia, Target identification

## Abstract

Dasatinib and radotinib are oral BCR-ABL tyrosine kinase inhibitors that were developed as drugs for the treatment of chronic myeloid leukemia. We report here that the c-KIT (CD117) targeting with dasatinib and radotinib promotes acute myeloid leukemia (AML) cell death, and c-KIT endocytosis is essential for triggering c-KIT-positive AML cell death by dasatinib and radotinib during the early stages. In addition, dasatinib and radotinib reduce heat shock protein 90β (HSP90β) expression and release Apaf-1 in c-KIT-positive AML cells. Finally, this activates a caspase-dependent apoptotic pathway in c-KIT-positive AML cells. Moreover, the inhibition of c-KIT endocytosis by dynamin inhibitor (DY) reversed cell viability and c-KIT expression by dasatinib and radotinib. HSP90β expression was recovered by DY in c-KIT-positive AML cells as well. Furthermore, the effect of radotinib on c-KIT and HSP90β showed the same pattern in a xenograft animal model using HEL92.1.7 cells. Therefore, dasatinib and radotinib promote AML cell death by targeting c-KIT. Taken together, these results indicate that dasatinib and radotinib treatment have a potential role in anti-leukemic therapy on c-KIT-positive AML cells.

## Introduction

Acute myeloid leukemia (AML) is one of the most difficult malignancies to cure. It is a heterogeneous collection of hematopoietic malignancies that can be classified into genetically distinct and prognostically relevant subtypes^1,2^. There are unmet needs in AML treatment because the current standard therapy is based on old chemotherapeutic regimens, which were established 3 decades ago. Although many molecular targets have been investigated, c-KIT (CD117) is a potential target in AML treatment. In particular, the proto-oncogene *c-KIT* is expressed in approximately 80% of AML cases, and its expression is a reliable molecular marker of poor prognosis in AML^3,4^. Moreover, *KIT* mutations are found in 25% to 30% of cases of core-binding factor (CBF)-AML, which is genetically expressed by the presence of t(8;21)(q22;q22) or inv(16)(p13;q22)^5^. In most studies, *KIT* mutations have been associated with poor clinical outcomes.

The molecular chaperone heat shock protein 90 (HSP90) is expressed abundantly in many cancers including solid tumors and hematological malignancies and facilitates the function of numerous oncoproteins^6,7^. Consequently, it has been well known that HSP90 is regarded a therapeutic target^8–10^. In addition, it plays a key role in assisting in the correct folding and functionality of its client proteins which include a variety of signal transducing molecules for cellular homeostasis, tumorigenesis, and others^6,8^. Especially, HSP90 is expressed in most AML specimens, at variable levels^11^. In this regard, HSP90 is another potential therapeutic target in AML.

Dasatinib is an FDA-approved small molecular compound that was developed for the treatment of chronic myeloid leukemia (CML) as a multi-targeted tyrosine kinase inhibitor (TKI)^12^. It inhibits the following kinases: BCR-ABL, SRC family, c-KIT and PDGFRβ^13–16^. Dasatinib is an important option for the treatment of patients with newly diagnosed chronic-phase CML (CP-CML) and for imatinib-resistant or -intolerant patients with chronic- or advanced-phase CML or Philadelphia-positive acute lymphoid leukemia^17^. Previously, we confirmed that dasatinib has an increased cytotoxicity against most types of AML cells^18^. And it inhibits proliferation and induces apoptosis in the AML cells^19^.

Another BCR-ABL1 TKI, radotinib was developed as a drug for the management of CP-CML in South Korea^20,21^. It is an effective inhibitor of native and kinase domain mutant BCR-ABL1^21^. We previously demonstrated that radotinib has increased cytotoxicity against all types of AML cells^22^. In addition, it inhibits AML cell proliferation through the activation of mitochondria-dependent apoptosis and the induction of the CDK inhibitors, p21 and p27^23^. Moreover, radotinib induces apoptosis in CD11b^+^ cells differentiated from AML cells^22^.

In these studies, we realized that of the AML cells, c-KIT-positive cells are more sensitive to dasatinib and radotinib. However, it was not clear what kind of signaling pathways are involved in cell death in c-KIT-positive AML. It is important to identify the signal molecules that are influenced and/or targeted by dasatinib and radotinib in AML cells as well as the underlying mechanisms.

## Results

### Dasatinib and radotinib induces high cytotoxicity in c-KIT-positive AML cells

**Figure 1:**
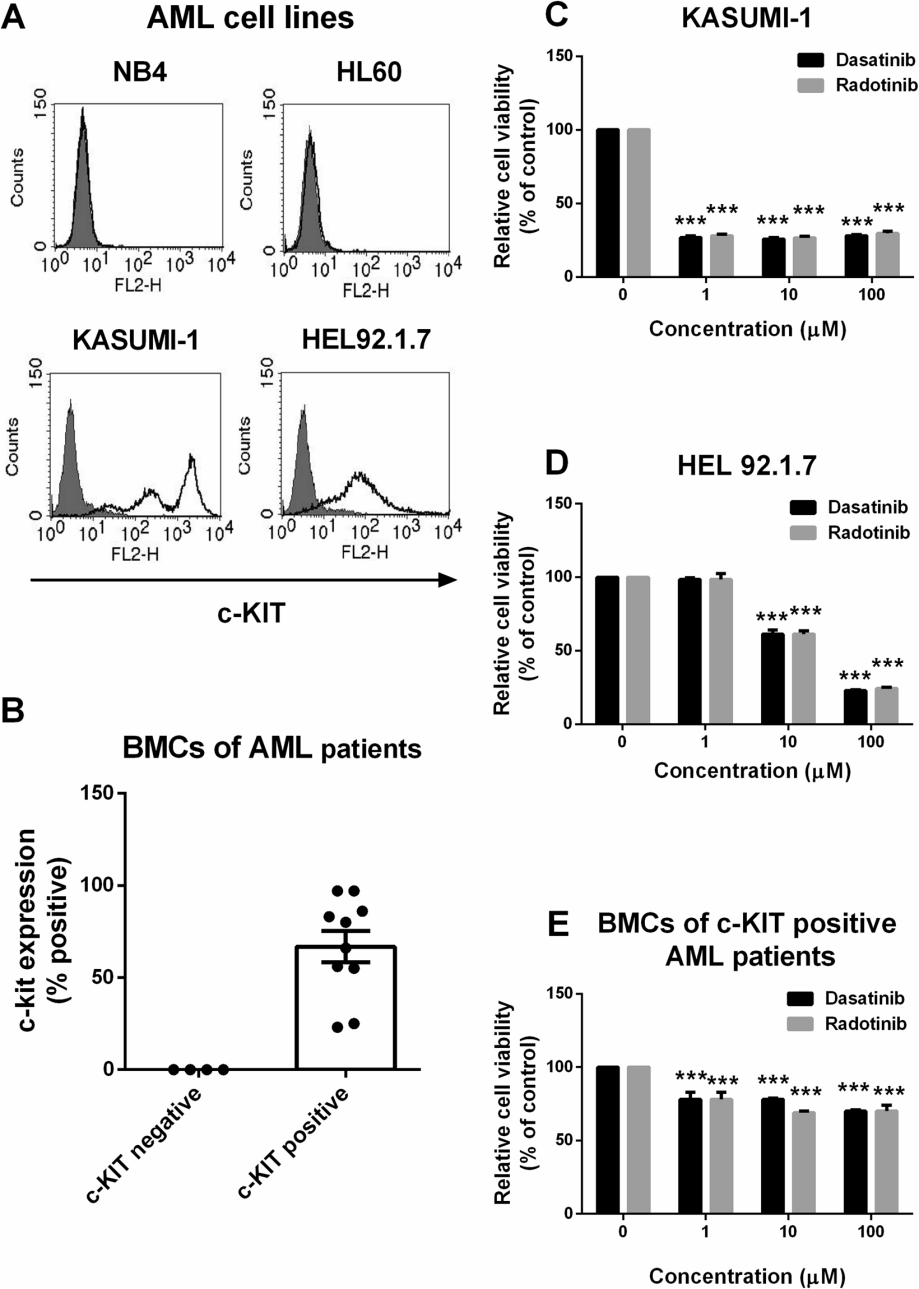
The effects of dasatinib and radotinib on the viability of c-KIT-positive AML cells. (**A**) The expression of c-KIT in AML cell lines. (**B**) The expression of c-KIT in bone marrow cells (BMCs) of AML patients (c-KIT-negative, n = 4; c-KIT-positive, n = 10). (**C**) Cell viability of KASUMI-1 cells. (**D**) Cell viability of HEL92.1.7 cells. (**E**) Cell viability of BMCs of c-KIT-positive AML patients. These data represent the means ± SEM. Significantly different from the control (*); ***p < 0.001. BMCs, bone marrow cells.

At first, we stained the cell surfaces of AML cell lines and BMCs from AML patients with c-KIT antigen. KASUMI-1 and HEL92.1.7 cells were found to be c-KIT-positive AML cell lines, but not NB4 and HL-60 cells (Fig. 1A). After the c-KIT-positive and negative BMCs from AML patients were chosen (c-KIT-positive samples, n = 10; c-KIT-negative samples, n = 4), the c-KIT antigen expression was confirmed on BMCs of AML patients (Fig. 1B, Supplementary Table 1). Thus, dasatinib and radotinib induced high cytotoxicity in c-KIT-positive AML cells, but the underlying mechanisms in these cells were not clear. The cytotoxic effect of dasatinib and radotinib were very similar on c-KIT-positive AML cells including KASUMI-1, HEL92.1.7 and BMCs, as shown Fig. 1C–E.

### Dasatinib and radotinib induce a reduction in c-KIT expression in KASUMI-1 and HEL92.1.7 cells

**Figure 2:**
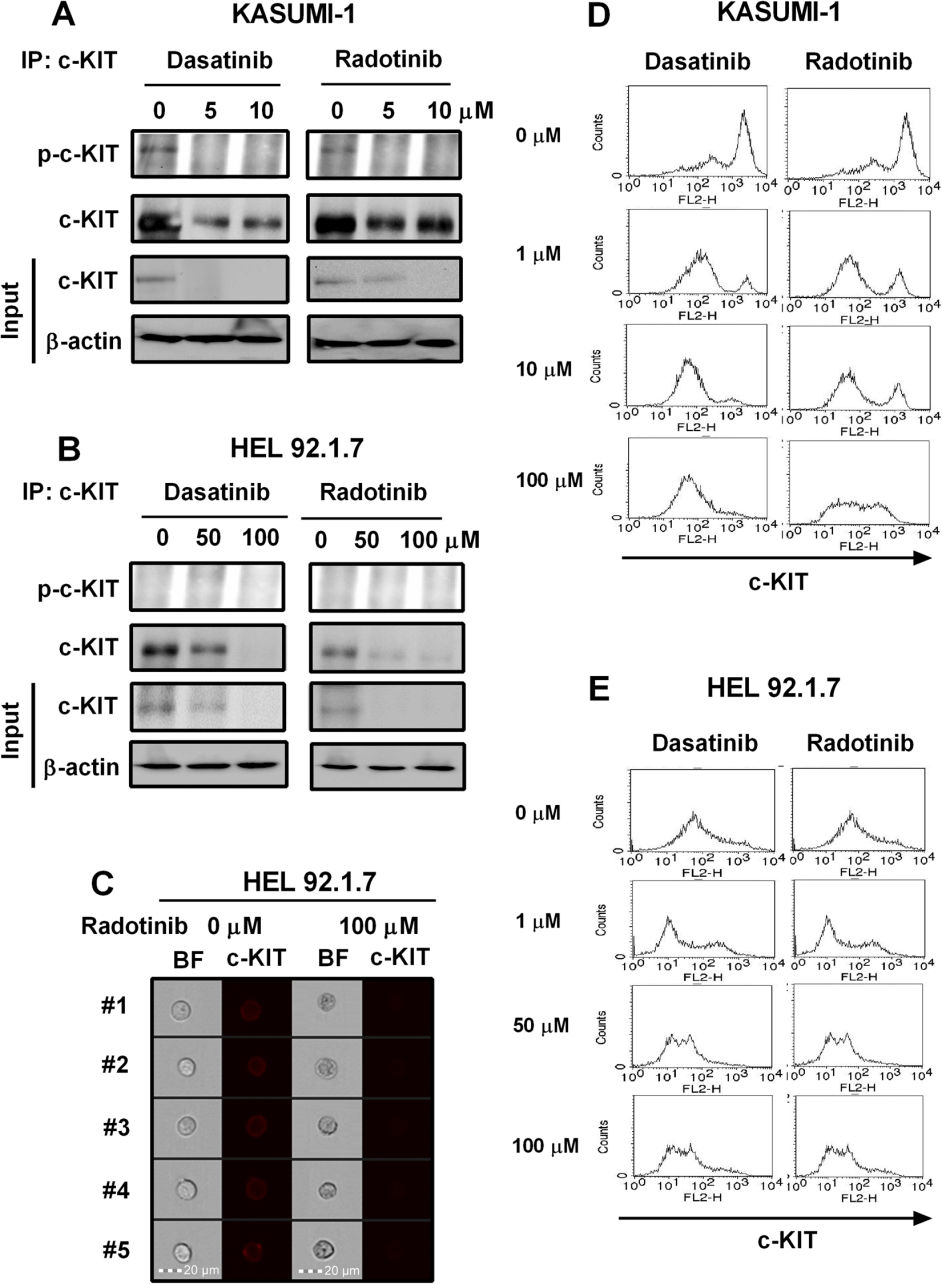
Dasatinib and radotinib inhibit c-KIT activity and expression in the c-KIT-positive cell lines, KASUMI-1 and HEL92.1.7. (**A**,**D**) The c-KIT activity and expression in KASUMI-1 cells. (**B**,**C**,**E**) The c-KIT activity and expression in HEL92.1.7 cells. BF, bright field.

As shown in Fig. 2A, dasatinib, and radotinib decreased not only the c-KIT activity but also c-KIT expression in KASUMI-1 cells which have a c-KIT activation mutation^24,25^. Also, dasatinib and radotinib down-regulated c-KIT expression in HEL92.1.7 cells, i.e., the human erythroleukemia cell line (Fig. 2B)^26,27^. Also, HEL92.1.7 cells showed decreased expression of cell surface c-KIT antigen by radotinib (image data), as shown Fig. 2C. Moreover, cell surface c-KIT antigen expression in both cell lines were dramatically reduced by dasatinib and radotinib, when analyzed via flow cytometry (Fig. 2D and E).

### C-KIT suppression by dasatinib and radotinib is essential for apoptosis of AML cells via activation of the caspase-dependent apoptotic pathway

We examined the relationship between cell viability and c-KIT expression in dasatinib- and radotinib-treated KASUMI-1 and HEL92.1.7 cells. We realized that cells that showed higher c-KIT expression had a higher cell survival rate (Supplementary Fig. 1).

**Figure 3:**
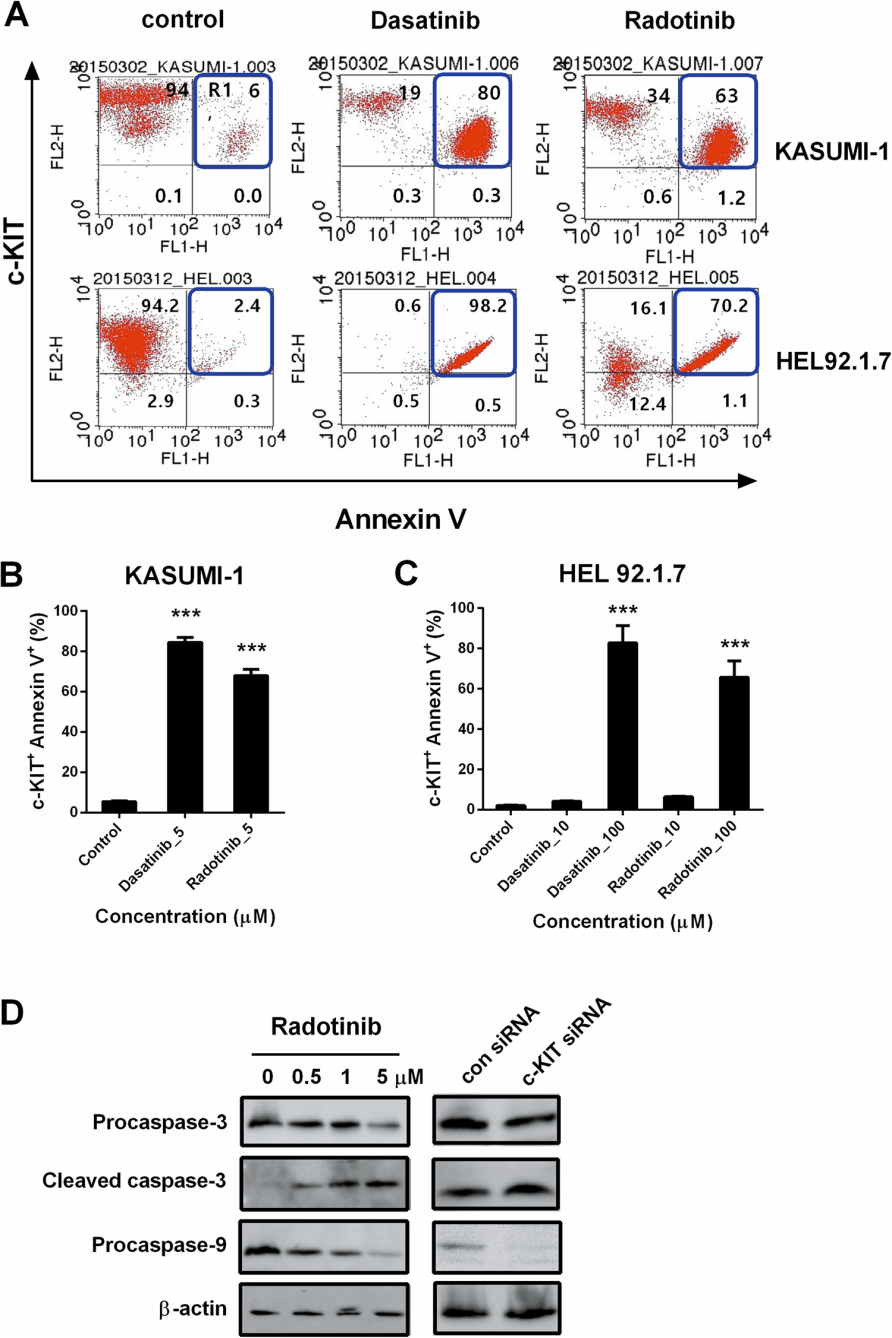
Dasatinib and radotinib induce expression of c-KIT^+^AnnexinV^+^ cells in AML cell lines, and activates the caspase pathway. (**A**) The expression of c-KIT^+^AnnexinV^+^ cells in KASUMI-1 and HEL92.1.7 cells. (**B**,**C**) Summary of the A. (**D**) The expression of procaspase-3, cleaved caspase-3 and procaspase-9 in KASUMI-1 by radotinib, control siRNA, and c-KIT siRNA treatment. β-actin levels were used as internal markers for loading variation. These data represent the means ± SEM. Significantly different from the control (*); ***p < 0.001. AML, acute myeloid leukemia.

**Figure 4:**
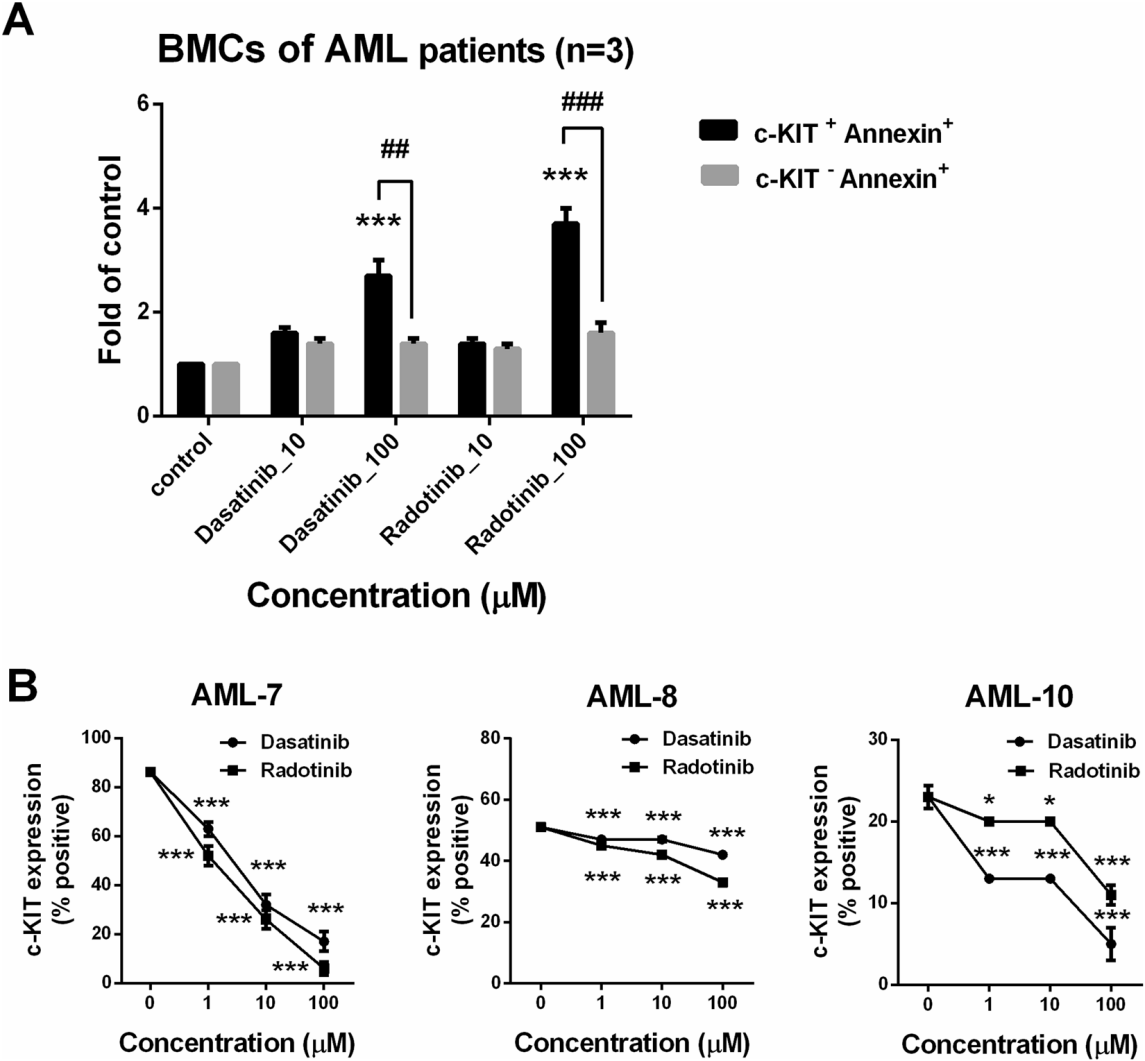
Dasatinib and radotinib induce expression of c-KIT^+^ Annexin V^+^ cells in BMCs of c-KIT-positive AML patients. (**A**) The expression of c-KIT^+^ Annexin V^+^ cells in BMCs of c-KIT-positive AML patients. (**B**) The c-KIT expression in BMCs of c-KIT-positive AML patients (AML-7, AML-8, and AML-10 in Supplementary Table 1) by dasatinib and radotinib treatment. These data represent the means ± SEM. Significantly different from the control (*); ***p < 0.001. AML, acute myeloid leukemia; BMCs, bone marrow cells.

To confirm the relationship between c-KIT expression and AML cell death with dasatinib and radotinib, we analyzed c-KIT, and Annexin V double positive cells by flow cytometry. The results showed that the number of Annexin V positive cells affected by dasatinib and radotinib were increased in the c-KIT-positive AML cell lines, KASUMI-1 and HEL92.1.7 cells, in a dose-dependent manner (Fig. 3A–C). Moreover, radotinib induced the activation of the caspase pathway in both cell lines (Fig. 3D and Supplementary Fig. 2). According to Supplementary Fig. 2A, the inhibition efficiency of c-KIT protein by c-KIT siRNA in HEL92.1.7 cells was over than 98%. As shown in Fig. 3D and Supplementary Fig. 2B, inhibition of c-KIT expression in KASUMI-1 and HEL92.1.7 cells markedly increased apoptotic signals. In regards these points, the induction of AML cell death was started by the suppression or degradation of c-KIT protein in the c-KIT positive AML cells. Furthermore, radotinib augmented the activation of the caspase pathway, and the status was very like that of c-KIT inhibition by c-KIT siRNA in both cell lines (Fig. 3D and Supplementary Fig. 2). Therefore, the results indicate that c-KIT suppression by dasatinib and radotinib is necessary for AML cell death via apoptotic pathway activation. In other words, c-KIT suppression by dasatinib and radotinib triggered AML cell death in c-KIT-positive AML cells. As shown Fig. 4, we also confirmed the effects of dasatinib and radotinib on the BMCs of AML patients. When we analyzed c-KIT, and Annexin V double positive cells via flow cytometry, these cells were significantly enhanced by dasatinib and radotinib in BMCs of patients with AML (Fig. 4A). Furthermore, dasatinib and radotinib induced c-KIT suppression on the BMCs of AML patients (Fig. 4B). These results showed the same pattern of responses for dasatinib and radotinib in both c-KIT-positive AML cell lines and c-KIT-positive BMCs from AML patients.

According to Supplementary Fig. 3, dasatinib and radotinib increased the expression of Cbl, as also known as E3 ligase, in a time-dependent fashion in KASUMI-1 and HEL92.1.7 cells, as well as apoptotic protease activating factor-1 (Apaf-1) in both cells. On the other hand, the expressions of poly (ADP-ribose) polymerase (PARP) and HSP90 proteins were significantly decreased in these cells. These results might be due to dasatinib- and radotinib-induced c-KIT suppression by lysosomal processing (Cbl induction), and activation of the apoptotic pathway in c-KIT-positive AML cells (PARP and caspase activation; Supplementary Fig. 2 and Fig. 3).

### C-KIT endocytosis by dasatinib and radotinib triggers death of c-KIT-positive AML cells

**Figure 5:**
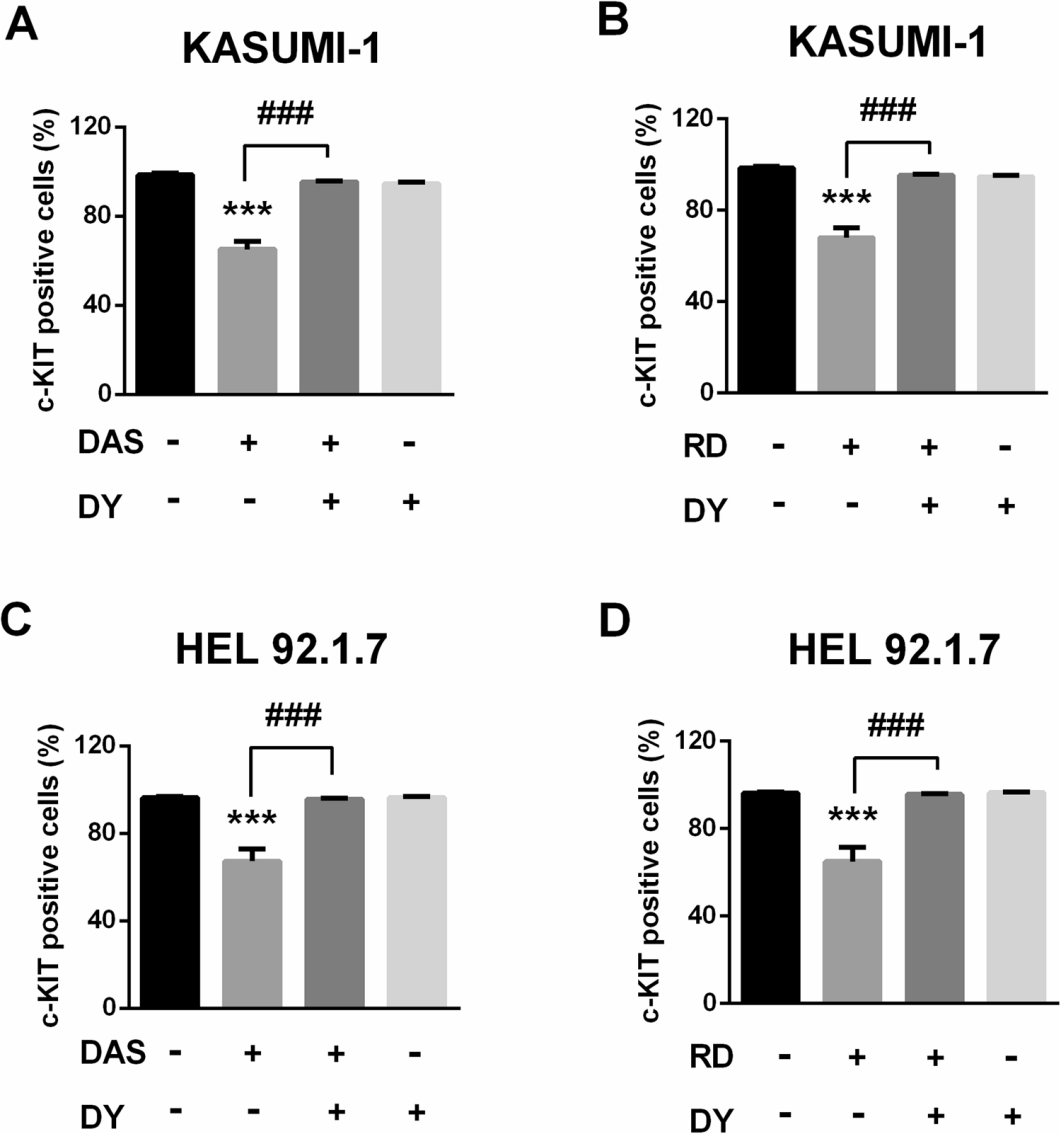
DY, dynamin inhibitor, recovers dasatinib or radotinib-induced c-KIT suppression in KASUMI-1 and HEL92.1.7 cells. (**A**,**B**) The expression of c-KIT in KASUMI-1 cells by dasatinib or radotinib, and DY treatment. (**C**,**D**) The expression of c-KIT in HEL92.1.7 cells by dasatinib or radotinib, and DY treatment. Cells were preincubated with dynamin inhibitor, DY (80 μM) for 2 h at 37 °C before the addition of dasatinib or radotinib. These data represent the means ± SEM. Significantly different from the DMSO-treated control group (*) or dasatinib/radotinib-treated group (#); ***,^###^ *p* < 0.001. DAS, dasatinib; RD, radotinib; DY, dynasore.

Generally, it has been well known that receptor-mediated endocytosis is mediated by clathrin-coated vesicles^28^. The clathrin-mediated endocytosis regulation is important because cell surface signaling is controlled by endocytic trafficking^29^, is essential for the rapid clearance of surface receptors. Then we assessed c-KIT suppression by dasatinib and radotinib in c-KIT-positive AML cells which are associated with clathrin-coated vesicles using DY, a potent inhibitor of dynamin GTPase, which is crucial for clathrin-coated vesicle formation^30^. In dasatinib- and radotinib-treated cells, the levels of cell viability and cell surface c-KIT expression were decreased, and these effects were abolished after treatment with DY (Supplementary Fig. 4 and Fig. 5). Thus, the reduction in cell surface c-KIT expression by dasatinib and radotinib is via a clathrin-mediated endocytosis in those cells. Therefore, c-KIT is endocytosed in a clathrin-mediated pathway by dasatinib and radotinib, reduces c-KIT expression, and induces cell death in c-KIT-positive AML cells. These results indicate that c-KIT internalization by dasatinib and radotinib triggers c-KIT-positive AML cell death.

### Dasatinib and radotinib reduce HSP90 activity and expression in c-KIT-positive AML cells

HSP90 is expressed abundantly in many cancers including solid tumors and hematological malignancies^6^. It plays a pivotal role in assisting in the correct folding and functionality of its client proteins which include a variety of signal transducing molecules^8^ At first, the mRNA expression of HSP90β and HSP70 were very affected by dasatinib and radotinib (Supplementary Fig. 5). It was one of the reason we focused on HSP90β in this study. According to a recent report, the relationship between c-KIT and HSP90β plays an important role in the cell death of AML^28^. So, we studied with more interest in HSP90β. Also, it has been reported that HSP90β and Apaf-1 have a high binding affinity in AML cells and gastrointestinal stromal tumor (GIST) cells, which are c-KIT-positive cells^13,31^. As shown in Supplementary Fig. 3, we confirmed that Apaf-1 was associated with dasatinib and radotinib-induced AML cell death, and its expression was increased by dasatinib and radotinib in AML cells. At the same time, it meant to started activation of apoptotic pathway.

**Figure 6:**
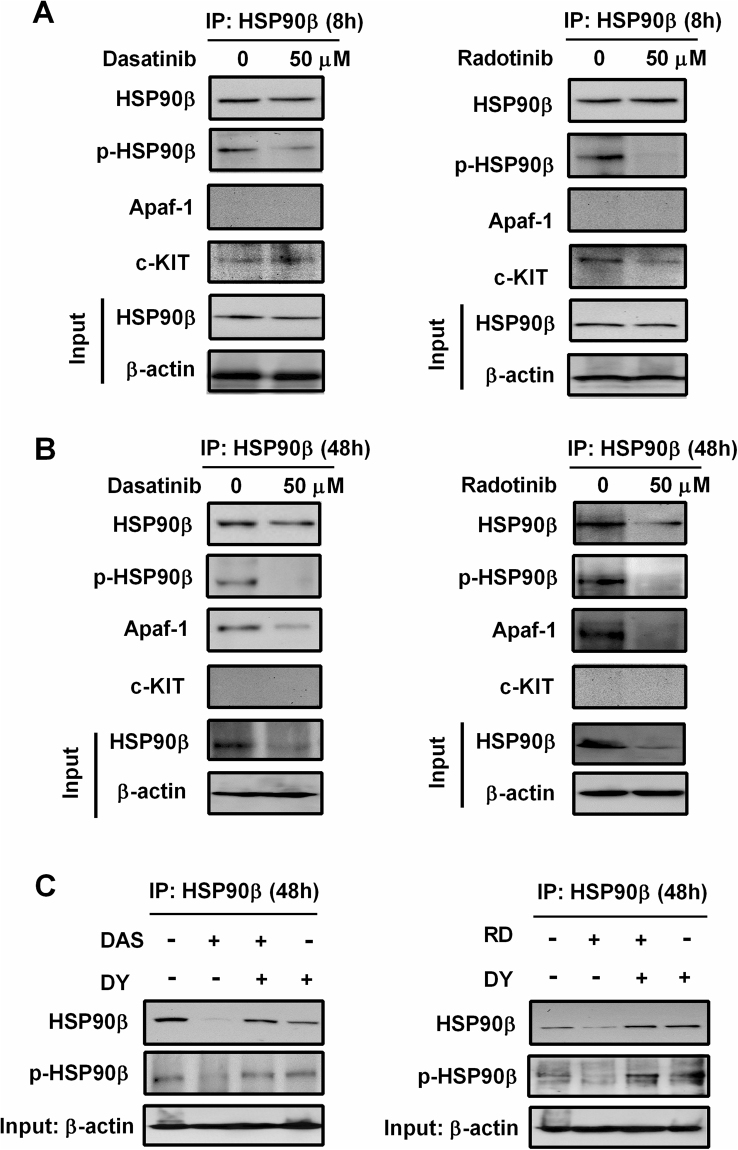
Dasatinib and radotinib inhibit HSP90β activity and expression in HEL92.1.7 cells, and regulate its binding with scaffold protein, Apaf-1 or c-KIT. (**A**) The HSP90β activity and expression by dasatinib or radotinib treatment at early time. After 8 h, HSP90β was immunoprecipitated from HEL92.1.7 samples treated as shown, and Western blotting shows phospho- and total HSP90β, Apaf-1, and c-KIT. (**B**) The HSP90β activity and expression by dasatinib or radotinib treatment at late time (48 h). (**C**) DY recovers dasatinib or radotinib-induced HSP90β suppression. DAS, dasatinib; RD, radotinib; DY, dynasore; HSP, heat shock protein.

The protein and mRNA expression of HSP90β were significantly decreased by dasatinib and radotinib in KASUMI-1 and HEL92.1.7 cells, in a time-dependent manner (Supplementary Figs 3 and 5). The effect on HSP70 was also of the same pattern in those cells (Supplementary Fig. 5). We also investigated the relationship among the HSP90β, Apaf-1, and c-KIT by co-immunoprecipitation and Western assay (Fig. 6), and the proposed pathways are presented in Supplementary Fig. 6 based on our results.

Earlier on (at 8 h), c-KIT bonded HSP90β which showed high levels of phosphorylation. The residue Y301 might have been the phosphorylation site of HSP90β, which was why we detected p-HSP90β by p-Tyr antibody^6^. Its activity was suppressed by dasatinib and radotinib in HEL92.1.7 cells (Fig. 6A and Supplementary Fig. 6A). And we found that dasatinib and radotinib significantly inhibited not only HSP90β phosphorylation, but also HSP90β expression in c-KIT-positive cell lines including HEL92.1.7 and KASUMI-1 cells at a later time (48 h), as shown in Fig. 6B, Supplementary Fig. 6B, and Supplementary Fig. 7A. Moreover, Apaf-1 was trapped by HSP90β, which had high levels of phosphorylation at 48 h in the cells of the control group. Dasatinib and radotinib significantly induced the degradation of HSP90β, and Apaf-1 was released from HSP90β and could trigger apoptosome formation in these cells.

Finally, dasatinib and radotinib activated apoptosis in c-KIT-positive cells. Therefore, the scaffolding of c-KIT, HSP90β, and Apaf-1 by dasatinib and radotinib played important roles in c-KIT-positive AML cell death (Fig. 6A and B). Furthermore, DY restored dasatinib- and radotinib-induced HSP90β suppression including expression and activity in HEL92.1.7 cells (Fig. 6C). These results indicate that dasatinib and radotinib function as an HSP90 inhibitor in AML cells regulated HSP90β activity and expression in c-KIT-positive AML cells that contributed to c-KIT-positive AML cell death (Supplementary Fig. 3, Supplementary Fig. 5 and Fig. 6).

### Radotinib inhibits c-KIT-positive AML cell growth *in vivo*

To evaluate the role of c-KIT and HSP90β in AML cell death *in vivo*, we engrafted HEL92.1.7 cells into nude mice. The results showed that the inhibition of c-KIT and HSP90β by radotinib led to a profound suppression of tumor growth, as evidenced by a significant reduction in tumor volume and weight (Supplementary Fig. 8A–C). Moreover, the expression of human CD45^+^c-KIT^+^ cells, human CD45^+^HSP90^+^ cells and human HSP90β mRNA in tumor tissues isolated from the mice were significantly decreased (Supplementary Fig. 8D–F).

## Discussion

C-KIT (CD117) is a tyrosine kinase receptor of the class III subfamily^32,33^. Generally, c-KIT in interaction with the ligand, stem cell factor, plays an important role in the survival, proliferation, differentiation, and functional activation of hematopoietic progenitor cells^1,33^. Therefore c-KIT is associated with AML cell survival; its expression signifies a poor prognosis in patients with AML^3,4^. Moreover, KIT mutations have been associated with poor outcomes in CBF-AML^5^.

Interestingly, the cytotoxicity of radotinib in c-KIT-positive BMCs were higher than that in c-KIT-negative BMCs from AML patients at low concentrations in our previous report^34^, and that was the motivation for this study. We are certain that c-KIT is involved in AML cell death. Moreover, dasatinib and radotinib showed a high cytotoxicity in c-KIT-positive AML cells^19,22,23^; but, it was not clear the underlying mechanisms, and we wanted to investigate this, as well as the signaling pathways involved.

First, dasatinib and radotinib induced c-KIT-positive AML cell death. For example, Annexin positive cells were increased, and caspase pathway were activated in those cells (procaspase-3 and -9 were significantly decreased; Figs 1, 3 and 4). At the same time, we observed that dasatinib and radotinib suppressed c-KIT expression in c-KIT-positive AML cells including KASUMI-1, HEL92.1.7, and BMCs from AML patients (Figs 2 and 4). After c-KIT endocytosis by dasatinib and radotinib, it might be degraded by lysosomal processing, and lead to activation of the apoptotic pathway (Annexin V positive cells and Apaf-1 expression were increased) in c-KIT-positive AML cells (Figs 3 and 4; Supplementary Fig. 2 and 3). The endocytosis of c-KIT by dasatinib and radotinib triggers c-KIT-positive AML cell death (Supplementary Fig. 4 and Fig. 5). Moreover, as shown in Fig. 3D and Supplementary Fig. 2B, inhibition of c-KIT expression in c-KIT-positive AML cells greatly increased cell death via activation of caspase-dependent apoptosis. Thus, cell death of the c-KIT positive AML cells was induced by the suppression of c-KIT protein. Therefore, c-KIT endocytosis/degradation by dasatinib and radotinib is required for c-KIT-positive AML cell death.

HSP90 is a chaperone protein that assists other proteins to fold properly, stabilizes proteins against heat stress, and aids in protein degradation. It also stabilizes many proteins required for tumor progression, which is why HSP90 inhibitors are being investigated as anti-cancer drugs for diverse cancers^6,7,9,35^. However, HSP90 inhibitors are not currently approved for cancer therapy^36^. Nowadays, the study of HSP90 inhibitors focuses on the preclinical activity profile in various tumors including AML, colorectal cancer, pancreatic cancer and breast cancer^37–42^. In our study, HSP90β proteins and mRNA were significantly decreased in dasatinib- and radotinib-treated c-KIT-positive AML cells (Supplementary Figs 3 and 5). Dasatinib and radotinib could regulate HSP90β activity and status of its binding protein (scaffolding protein; Fig. 6, Supplementary Figs 6 and 7A). These results indicate that dasatinib and radotinib function as a HSP90 inhibitor in AML cells. Moreover, HSP70 proteins and their activity were significantly decreased by radotinib in HEL92.1.7 cells (Supplementary Fig. 7B). Interestingly, recent studies have shown that AML treatment were studies by HSP90 or HSP70 inhibitors. In addition, combination therapy using conventional anticancer drugs and HSP90 inhibitors are actively underway^43–47^. So, we are preparing for the study about the combination effects of radotinib and conventional chemotherapeutic agents.

We have confirmed that radotinib inhibits c-KIT-positive AML cell lines using HEL92.1.7 cells in the xenograft tumor mouse model (Supplementary Fig. 8). Furthermore, the effects of radotinib on c-KIT and HSP90β were the same in both *in-vitro* and *in vivo* studies.

According to a recent report that FLT3 inhibitor have recently been adopted for standard chemotherapy^48^. We think that this is a very big achievement. Because the current standard therapy is based on old chemotherapeutic regimens, which were established 3 decades ago. Thus, it is so difficult to develop the new standard treatment. The FLT3 positive AML patients are 10% of all AML patients^48^. On the other hand, c-KIT positive AML patients accounted for 70-80% of all AML patients^3,4^. In this regard, the development of dasatinib or radotinib as a treatment for AML is very important. Therefore, we are researching the possibility of using clinical through experiments about the combination effects of radotinib and conventional chemotherapeutic agent, Ara C.

Lastly, we summarized the proposed signaling pathway of dasatinib and radotinib in c-KIT-positive AML cells in Supplementary Fig. 9. Therefore, dasatinib and radotinib may play an important role in the treatment of c-KIT-positive cancers including AML, prostate cancer, etc. In conclusion, these promising data suggest that dasatinib and radotinib are potential target agents for the treatment of c-KIT-positive AML by their regulation of HSP90β activity and expression.

## Materials and Methods

### Reagents

All reagents were obtained from Sigma-Aldrich (St. Louis, MO, USA) unless otherwise indicated. Anti-human c-KIT (CD117)-PE antibody, isotype control (mouse Ig G-PE), and Apoptosis Detection Kit I were purchased from BD Biosciences (San Jose, CA, USA). The CellTiter 96 AQueous One Solution Cell Proliferation Assay (MTS) was purchased from Promega (Madison, WI, USA). Fetal bovine serum (FBS), RPMI-1640 medium, and penicillin-streptomycin solution were purchased from Gibco BRL (Grand Island, NY, USA). All antibodies for western blot were purchased from Cell Signaling Technology (Beverly, MA, USA), and antibodies for immunoprecipitation against c-KIT and HSP90β were purchased from Santa Cruz Biotechnology (Santa Cruz, CA, USA).

### Patient samples

All patients had been newly diagnosed with AML (n = 14) at Ulsan University Hospital, Ulsan, South Korea, as described in Supplementary Table 1. The bone marrow samples were collected prior to their first round of chemotherapy.

### Ethics statement

All experiments were performed in accordance with the relevant guidelines and regulations. All patients provided written informed consent before the commencement of the study. The study protocol and patient consent form and information were approved by the Ethics Committee and Institutional Review Board of the Ulsan University Hospital (UUH-IRB-11-18). Mice experiments were performed in accordance with the relevant guidelines and regulations established by the ethical guidelines and regulations of the Korean Association for Laboratory Animals. And all experimental procedures were approved by the Institutional Animal Care and Use Committee of the Ulsan University of Korea (Approval No. 0117-07).

### Isolation and culture of patients’ cells

The patients’ cells were isolated by the density gradient method, as previously described^18^. In brief, bone marrow cells (BMCs) were isolated via density gradient centrifugation at 400 × *g* using Lymphoprep (Axis-Shield, Oslo, Norway). They were washed with phosphate-buffered saline (PBS) and cultured in the RPMI-1640 medium with 10% FBS and 1% penicillin-streptomycin solution in a 5% CO_2_ humidified atmosphere at 37 °C.

### Cell culture

The human AML cell lines in this study were grown as suspension cultures in 100-mm culture dishes in RPMI-1640 medium with 10% FBS (or 20% FBS for the Kasumi-1 cell line) and a 1% penicillin-streptomycin solution in a 5% CO_2_ humidified atmosphere at 37 °C, as previously described^22^.

### Cell surface staining of c-KIT

Cell surface staining was performed with anti-human c-KIT (CD117)-PE and isotype control mAb (mouse IgG-PE), as previously described^22^. The cells were then analyzed using a FACSCalibur flow cytometer and CellQuest Pro software (BD Bioscience). In some experiments, we analyzed the stained cells with a FlowSight^®^ cytometer and IDEAS software for image data.

### Detection of c-KIT^+^Annexin V^+^ cells

The cells were incubated with different concentrations of dasatinib and radotinib for 72 h at 37 °C, then harvested and washed twice with the FACS buffer (PBS containing 0.3% bovine serum albumin and 0.1% NaN_3_). First, the cells were stained with an anti-human c-KIT-PE at 4 °C for 30 min. After incubation, they were washed twice with the FACS buffer. Then the cells were incubated with Annexin V-FITC from the Apoptosis Detection Kit I at 4 °C for 30 min.

### Cell viability assay (MTS assay)

Cell viability assay was performed using the CellTiter 96 solution, as previously described^22,23^. In some experiments, cells were preincubated with the dynamin inhibitor, dynasore (DY, 80 μM) for 2 h at 37 °C prior to the addition of dasatinib or radotinib.

### Transfection of c-KIT siRNA

siRNA for c-KIT (cat No. E-003150-00-0005) and Accell Non-Targeting Control siRNA (cat No. D-001910-01-0005) were purchased from Dharmacon. The cells were transfected with siRNA using the Accell System (Dharmacon), cultured for 48 h, and then used in the western blot analysis. To investigate for efficiency of transfection, we used the Accell Green Non-targeting Control siRNA. The efficiency of transfection was over 95% in the cells. More than 98% of the c-KIT proteins were gone by siRNA.

### Western blot analysis and immunoprecipitation

For immunoprecipitation experiments, the cells were lysed as previous described^22^. KASUMI-1 and HEL92.1.7 cells (1 × 10^7^ cells/ml) were incubated with dasatinib or radotinib for 48 h and lysed in radioimmunoprecipitation assay buffer for 30 min at 4 °C. Equal amounts of protein were pre-cleared with Protein G beads. The beads were pelleted and the supernatant incubated with appropriate antibodies and beads overnight. Equal quantities of solubilized protein were resolved on 10% SDS-PAGE gel as previously described^22^. In some experiments, cells were preincubated with the DY (80 μM) for 2 h at 37 °C prior to the addition of dasatinib or radotinib.

### Trypan blue exclusion assay

The cells were incubated with 5 μM dasatinib and radotinib for 0, 8, 24 and 48 h at 37 °C, inoculated at a density of 10 × 10^4^ cells in each concentration of the drugs, and grown for 48 h. The grown cells were then harvested, and trypan blue was added to the cell suspension to a final concentration of 0.04%. Cells that excluded trypan blue (viable cells) were counted under the microscope with a hemocytometer. Each test was repeated a minimum of four times. In some experiments, cells were preincubated with 80 μM of DY for 2 h at 37 °C prior to the addition of dasatinib or radotinib.

### Xenograft animal model

Specific-pathogen-free five-week-old athymic nude male mice were purchased from Koatech (Pyeongtaek, Korea), and kept in a clean environment of the Ulsan University of Korea. HEL92.1.7 cells were injected subcutaneously into the right flank of the mice. The tumor’s maximal length and width were measured once a week using a digital caliper, and the tumor volume (V) calculated using the following formula: V = (length × width^2^) × 0.5. The mice were sacrificed on days 30–32 following tumor cell implantation. The body weight of the tumor-bearing mice did not change significantly during the duration of study. The tumors were excised and weighted, and each tumor tissue homogenized for the preparation of cell samples for several analyses.

### Statistics

The data presented here represent the means ± standard error of mean (SEM) of at least three independent experiments. All values were evaluated by a one-way analysis of variance followed by Turkey range tests implemented in GraphPad Prism 6.0 (GraphPad Software, Inc., La Jolla, USA). Differences were considered significant at *p* < 0.05. Each treatment was assayed in triplicate.

## Electronic supplementary material

Supplementary Information
