# Author Correction: Targeting c-KIT (CD117) by dasatinib and radotinib promotes acute myeloid leukemia cell death

**DOI:** 10.1038/s41598-021-02577-5

**Published:** 2021-11-26

**Authors:** Sook-Kyoung Heo, Eui-Kyu Noh, Jeong Yi Kim, Yoo Kyung Jeong, Jae-Cheol Jo, Yunsuk Choi, SuJin Koh, Jin Ho Baek, Young Joo Min, Hawk Kim

**Affiliations:** 1 Biomedical Research Center, Ulsan University Hospital, University of Ulsan College of Medicine, Ulsan, 682-060 Republic of Korea; 2 Department of Hematology and Oncology, Ulsan University Hospital, University of Ulsan College of Medicine, Ulsan, 682-714 Republic of Korea; 3 Division of Hematology, Gachon University Gil Medical Center, Gachon University College of Medicine, 21 Namdong-daero 774beon-gil, Incheon, 21565 Republic of Korea

Correction to: *Scientific Reports* 10.1038/s41598-017-15492-5, published online 10 November 2017

This Article contains errors.

As a result of errors during figure assembly Fig 6B Radotinib/c-kit image is a duplication of Fig 6A Radotinib/Apaf-1 image, and Fig S2B Radotinib PARP image is a duplication of Fig S3 HEL 92.1.7 PARP image.

The corrected Figure 6 and Figure S2B are shown below as Figure 1 and Figure 2.

**Figure 1:**
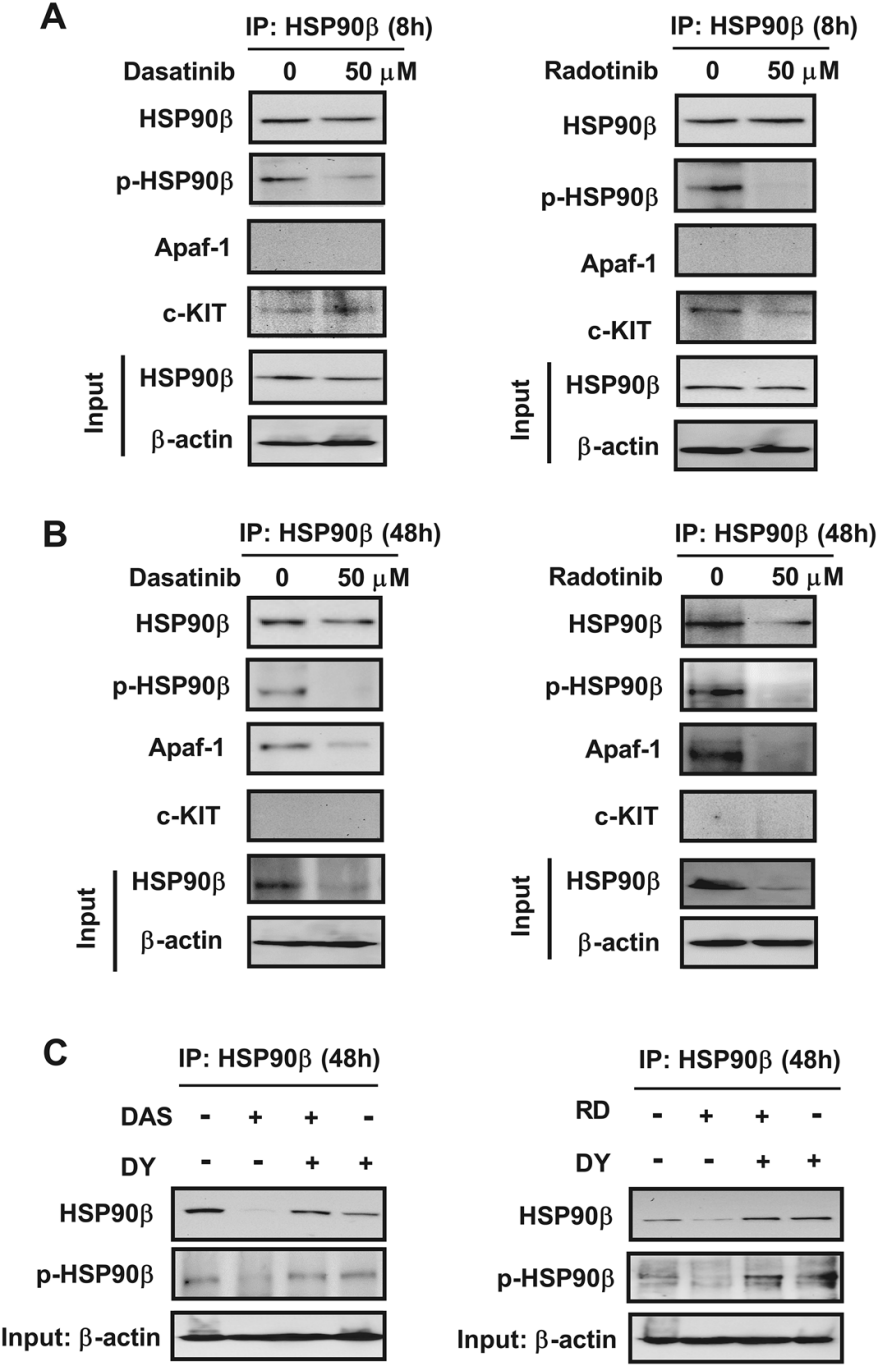
Corrected version of Figure 6.

**Figure 2:**
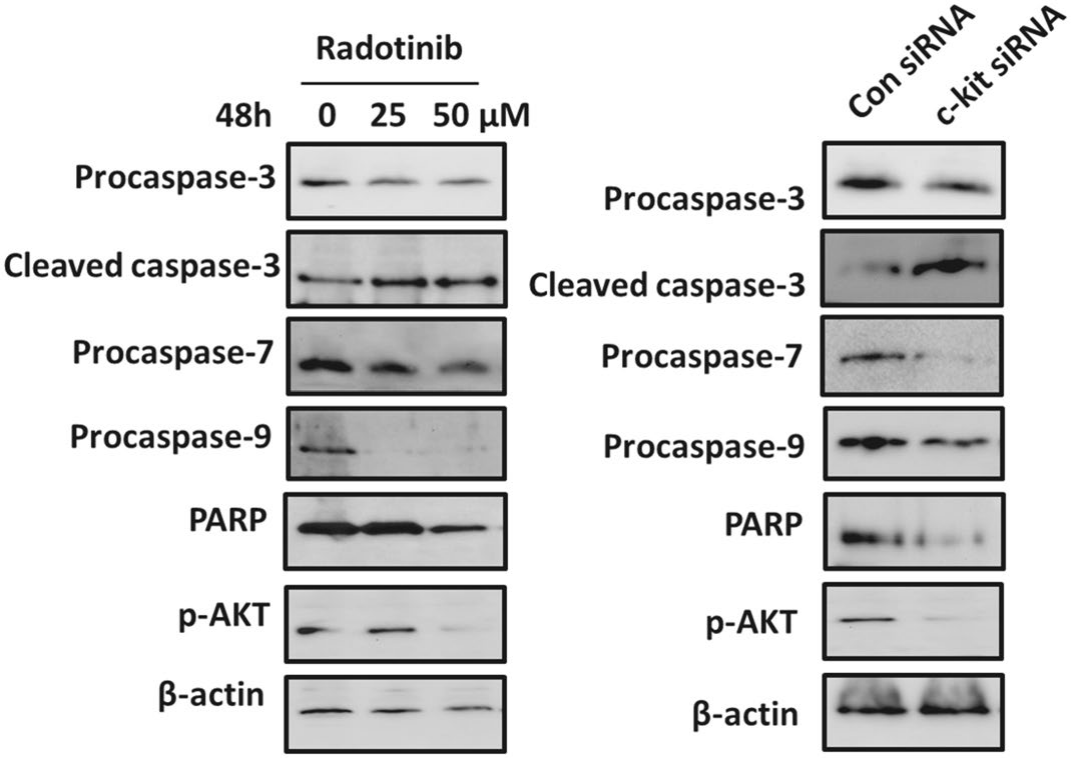
Corrected version of Figure S2.

Additionally, original images of the membranes showing Radotinib/Apaf-1, Radotinib/c-kit, and Radotinib/PARP results are shown below as Figure 3, 4, and 5, respectively.

**Figure 3:**
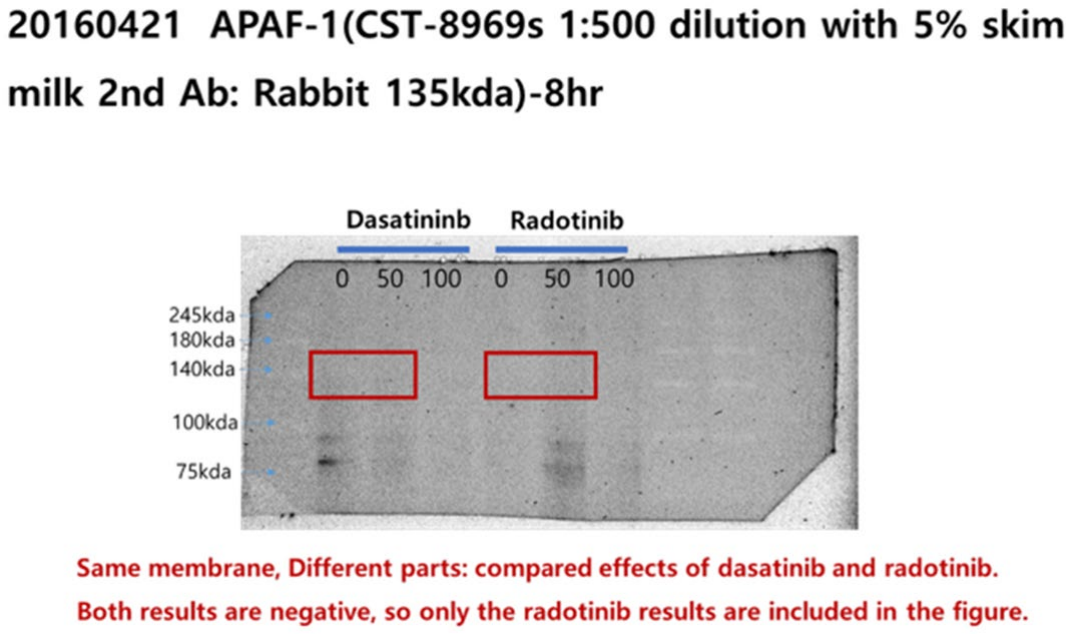
Original image of the membranes showing Radotinib/Apaf-1, corresponding to Fig 6A.

**Figure 4:**
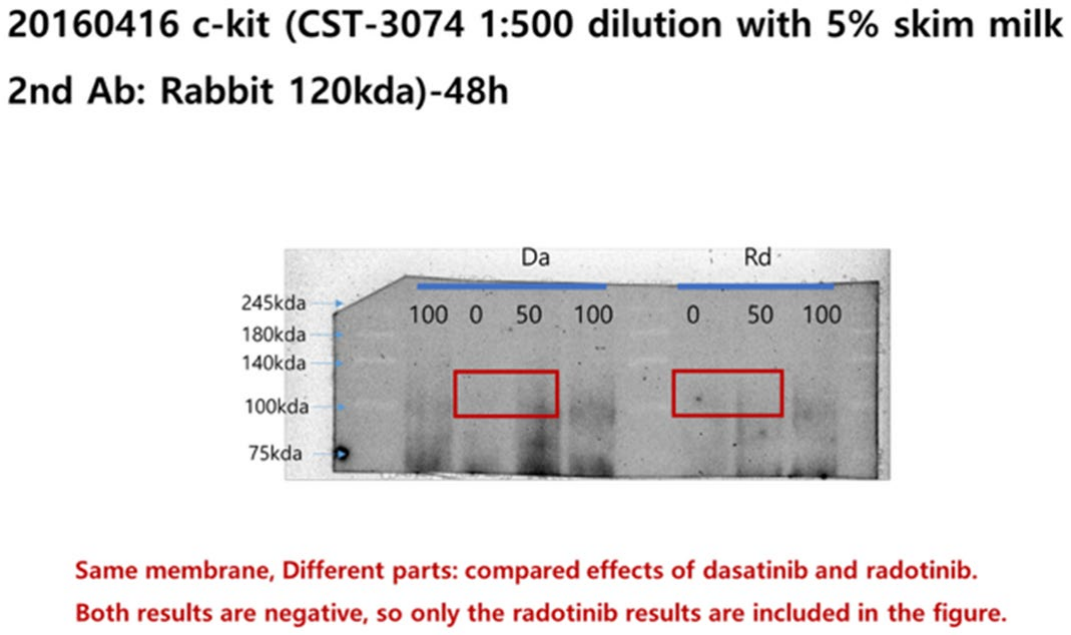
Original image of the membranes showing Radotinib/c-kit, corresponding to Fig 6B.

**Figure 5:**
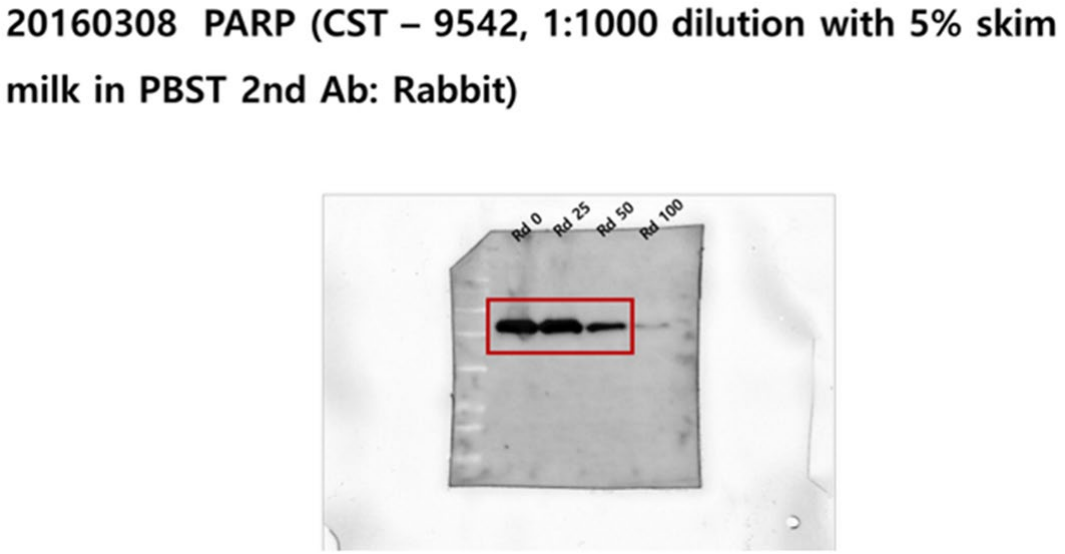
Original image of the membranes showing Radotinib/PARP, corresponding to Fig S2B.

The conclusions of the Article are not affected by these changes.

