# Supplementary material for: Targeting c-KIT (CD117) by dasatinib and radotinib promotes acute myeloid leukemia cell death
Source: Sci Rep. 2017 Nov 10;7:15278. doi: 10.1038/s41598-017-15492-5 (PMC5681687; doi:10.1038/s41598-017-15492-5)
Supplement: Supplementary file 1 — Supplementary Information [file 41598_2017_15492_MOESM1_ESM.pdf]

Supplementary Materials

Targeting c-KIT (CD117) by dasatinib and radotinib promotes acute myeloid leukemia cell death

Sook-Kyoung Heo, Eui-Kyu Noh, Jeong Yi Kim, Yoo Kyung Jeong, Jae-Cheol Jo, Yunsuk Choi, SuJin Koh, Jin Ho Baek, Young Joo Min, Hawk Kim

Supplementary Table 1. Information of AML patients.

UPN	Sex	Age	Disease	Cell source	c-KIT (%)	Blast (%)	Karyotype
1	F	72	AML, minimal differentiation	BM	97	93.5	46,XX[30]
2	M	75	AML, minimal differentiation	BM	97	91.5	46,XY[20]
3	M	16	AML with maturation	BM	80	66	46,XY[30]
4	F	69	AML, minimal differentiation	BM	86	93	46,XX[20]
5	M	52	Acute erythroid leukemia	BM	56	15.4	46,XY[20]
6	M	81	Acute myeloblastic leukemia with maturation	BM	66	45.5	46,XY,+1,der(1;7)(q10;p10)[18]/46,XY[2]
7	F	73	AML, minimal differentiation	BM	83	80	46,XX[7]
8	M	31	Acute myeloid leukemia without maturation	BM	55	73	46,XY[20]
9	M	49	leukemia without maturation	BM	25	34.5	46,XY[20]
10	M	57	Acute erythroid leukemia	BM	23	40.5	46,XY[20]
11	F	76	Acute myelomonocytic leukemia	BM	0	21	46,XX[6]
12	F	67	AML with maturation	BM	0	82.5	46,XX[8]
13	M	76	AML with maturation	BM	0	40	46,XY[20]
14	F	37	AML with maturation	BM	0	45	46,XX[25]

AML, acute myeloid leukemia; BM, bone marrow.

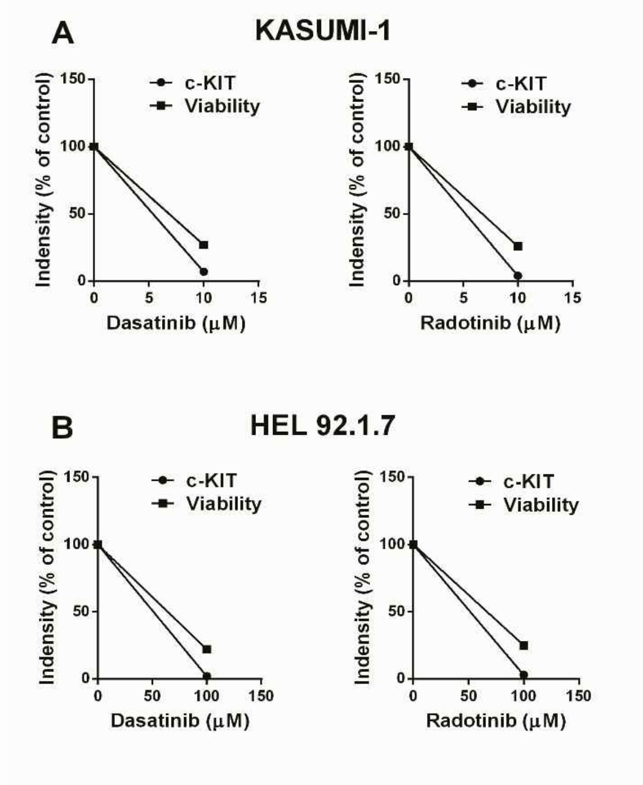

Supplementary Fig. 1. Correlation of cell viability and c-KIT expression in dasatinib and radotinib-treated AML cells. (A) KASUMI-1 cells. (B) HEL92.1.7 cells.

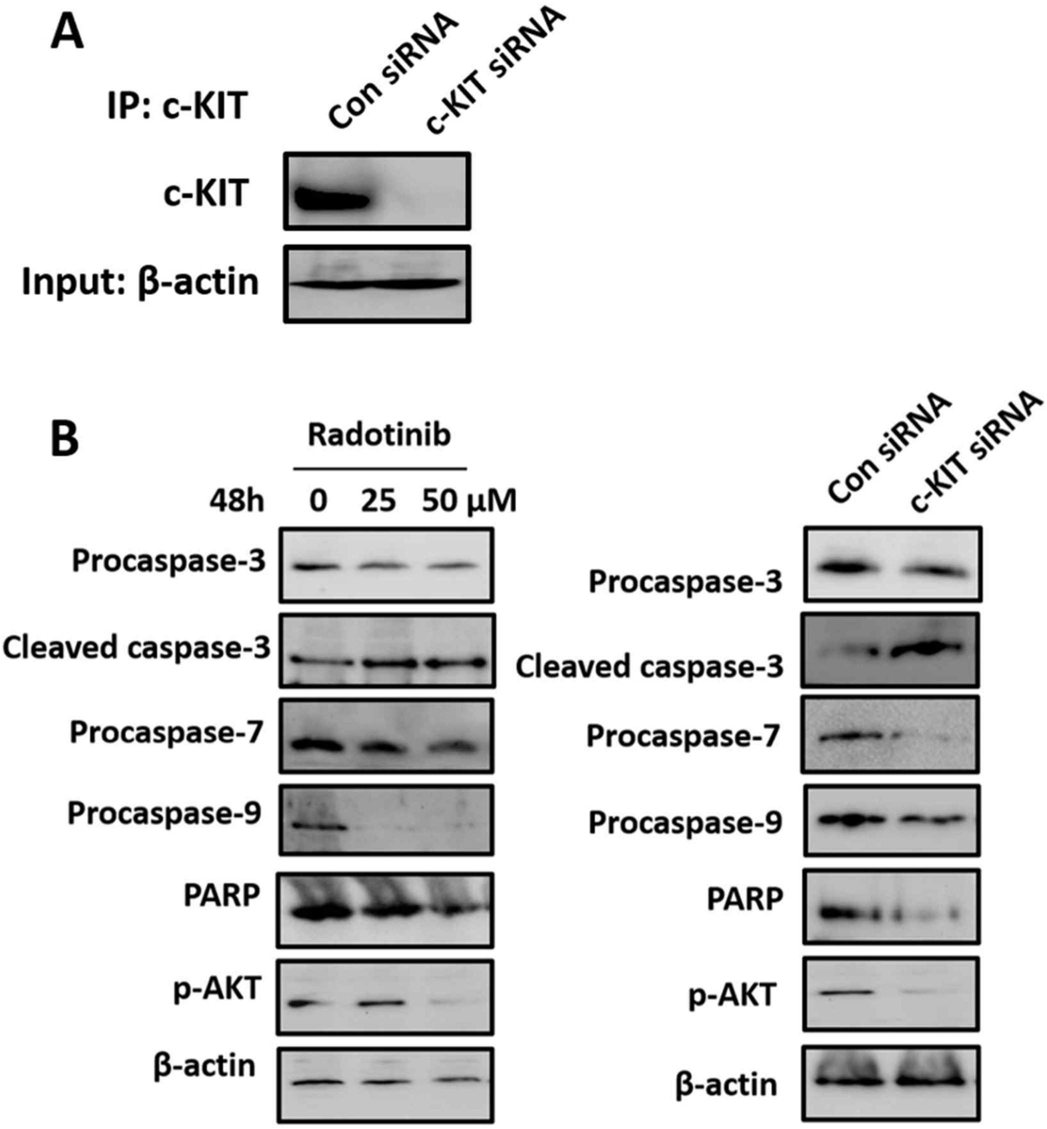

Supplementary Fig. 2. (A) Inhibition efficiency of c-KIT protein by c-KIT siRNA in HEL92.1.7 cells. (B) The expression of procaspase-3, cleaved caspase-3, procaspase-7, procaspase-9, PARP, and p-AKT in HEL 92.1.7 cells by radotinib, control siRNA and c-KIT siRNA treatment. β-actin levels were used as internal markers for loading variation. Each treatment was assayed in triplicate.

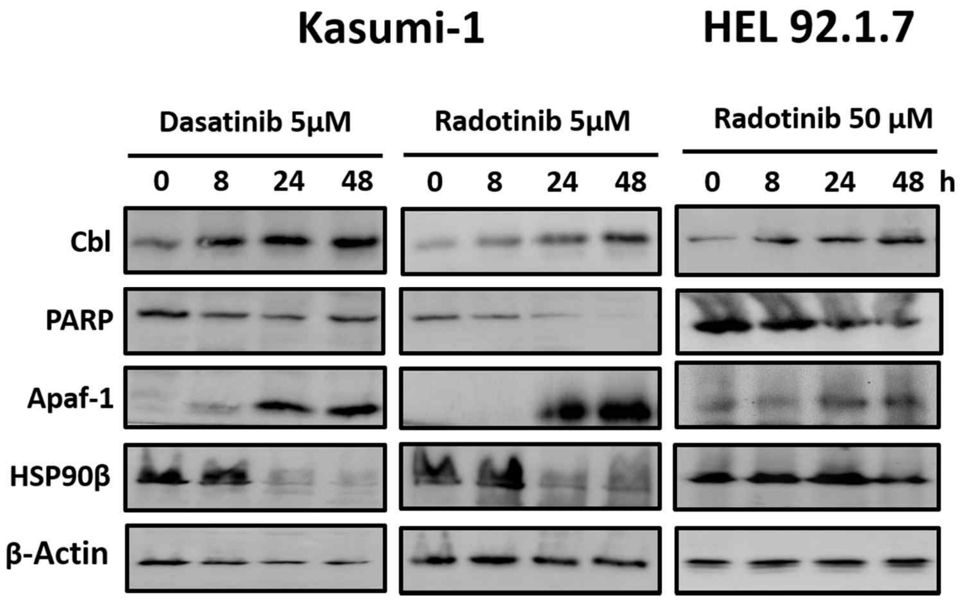

Supplementary Fig. 3. The expression of Cbl, PARP, Apaf-1 and HSP90β in KASUMI-1 and HEL92.1.7 cells by dasatinib or radotinib treatment for the indicated times. β-actin levels were used as internal markers for loading variation. Each treatment was assayed in triplicate.

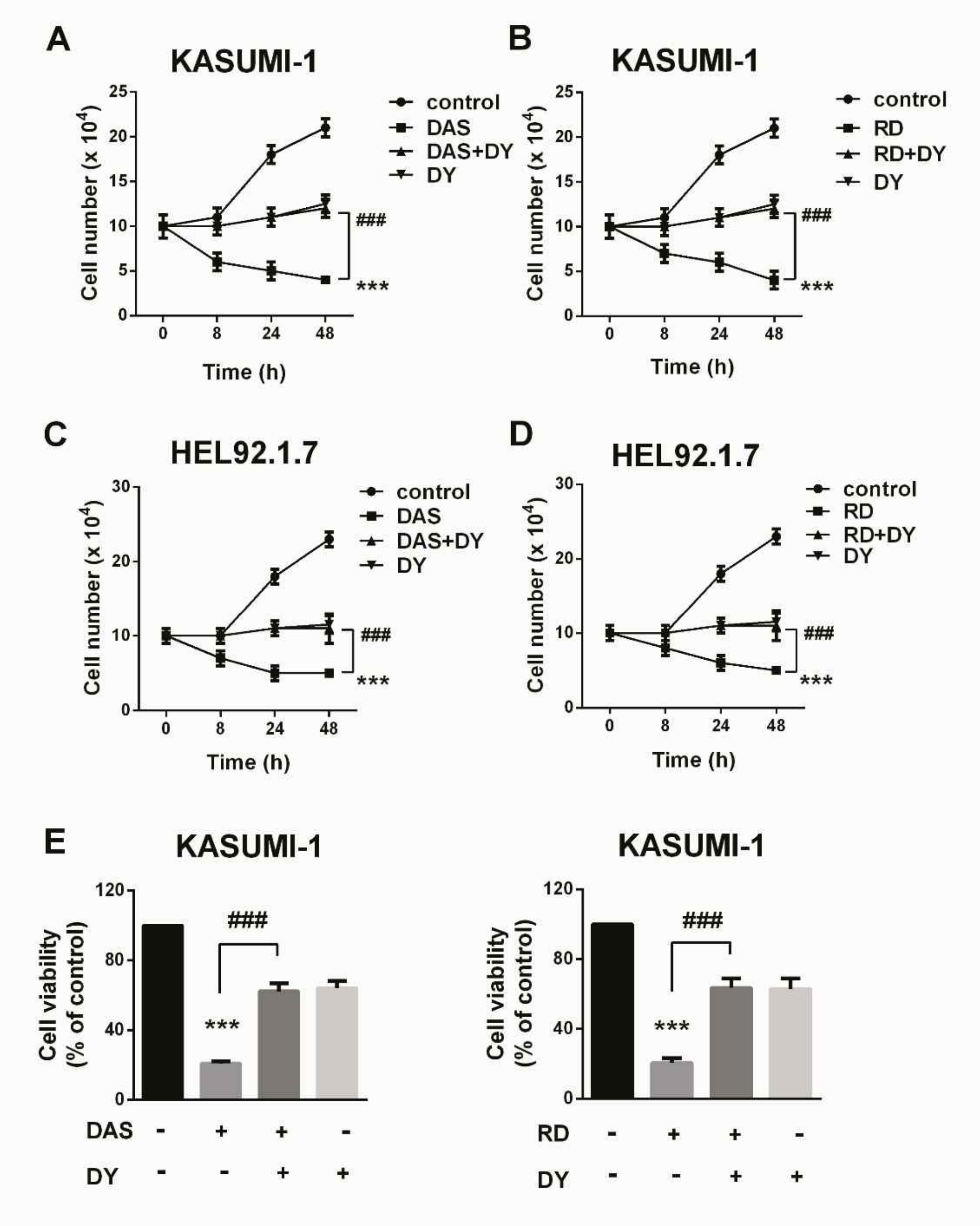

Supplementary Fig. 4. DY, dynamin inhibitor, recovers dasatinib or radotinib-induced AML cell death. (A, B) The cell numbers of KASUMI-1 by dasatinib or radotinib treatment. (C, D) The cell numbers of HEL92.1.7 by dasatinib or radotinib treatment. (E, F) The cell viability of KASUMI-1 by dasatinib or radotinib treatment. Cells were preincubated with dynamin inhibitor, DY (80 μM) for 2 h at 37°C before the addition of dasatinib or radotinib. These data represent the means ± SEM. Significantly different from the DMSO-treated control group (*) or dasatinib/radotinib-treated group (#); ***, ###, $p < 0.001$. DAS, dasatinib; RD, radotinib; DY, dynasore.

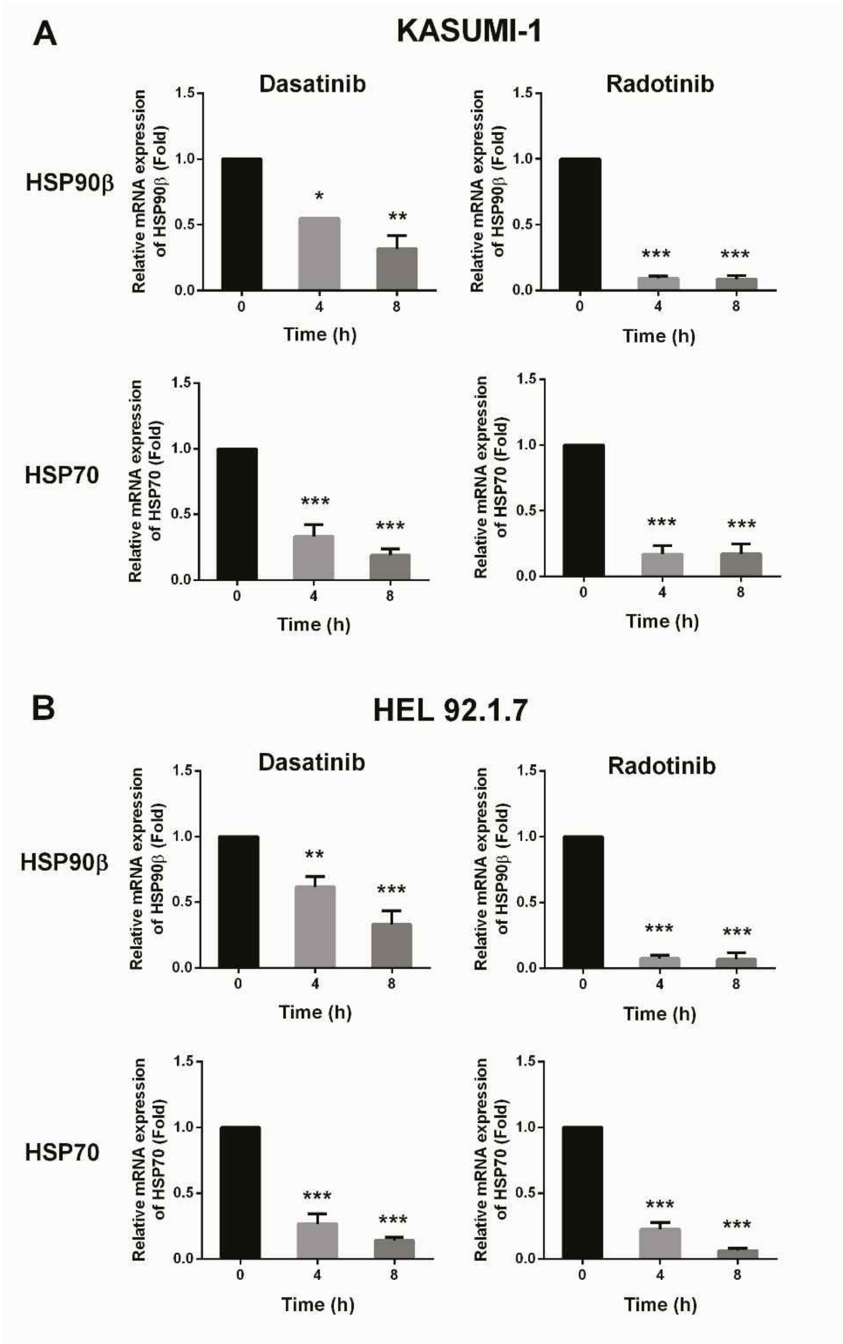

Supplementary Fig. 5. Dasatinib and radotinib inhibit HSP90β and HSP70 mRNA expression time-dependently in the c-KIT-positive cell lines, KASUMI-1 (A) and HEL92.1.7 cells (B). These data represent the means ± SEM. Significantly different from the control (*); *, $p < 0.05$; **, $p < 0.01$; ***, $p < 0.001$.

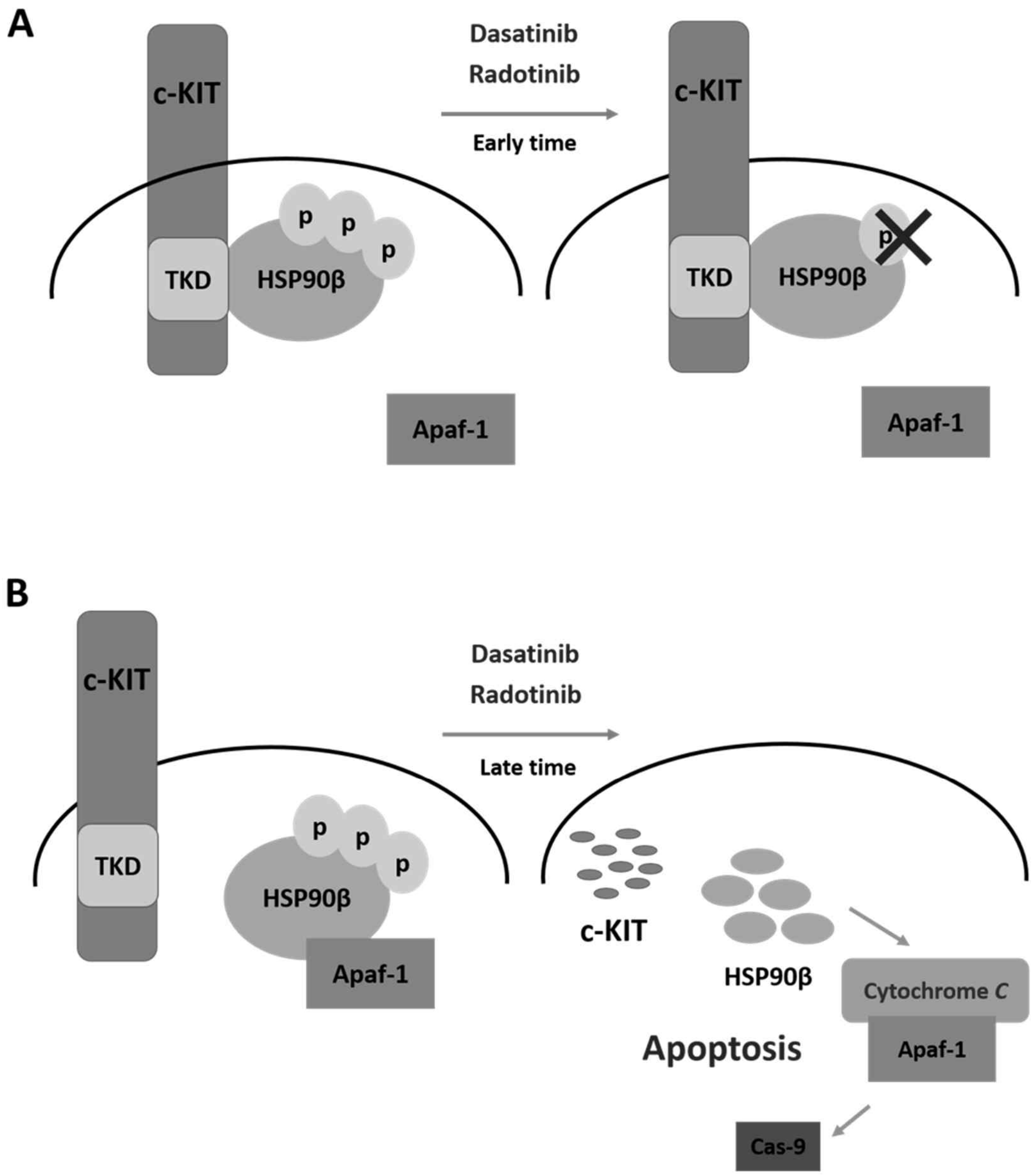

Supplementary Fig. 6. Proposed pathway of the study. Dasatinib and radotinib significantly inhibited not only HSP90β phosphorylation at early time (8 h), but also c-KIT and HSP90β expression at late time (48 h) in c-KIT-positive two cell lines including HEL92.1.7 and KASUMI-1 cells. Moreover, Apaf-1 was trapped by HSP90β, which was high levels of phosphorylation at 48 h in the cells of control group. Then dasatinib and radotinib significantly induced degradation of HSP90β, and Apaf-1 was released against HSP90β. Additionally, it could trigger to make the apoptosome formation in these cells. Finally, dasatinib and radotinib activated c-KIT-positive cells prone to be died via activation of apoptosis.

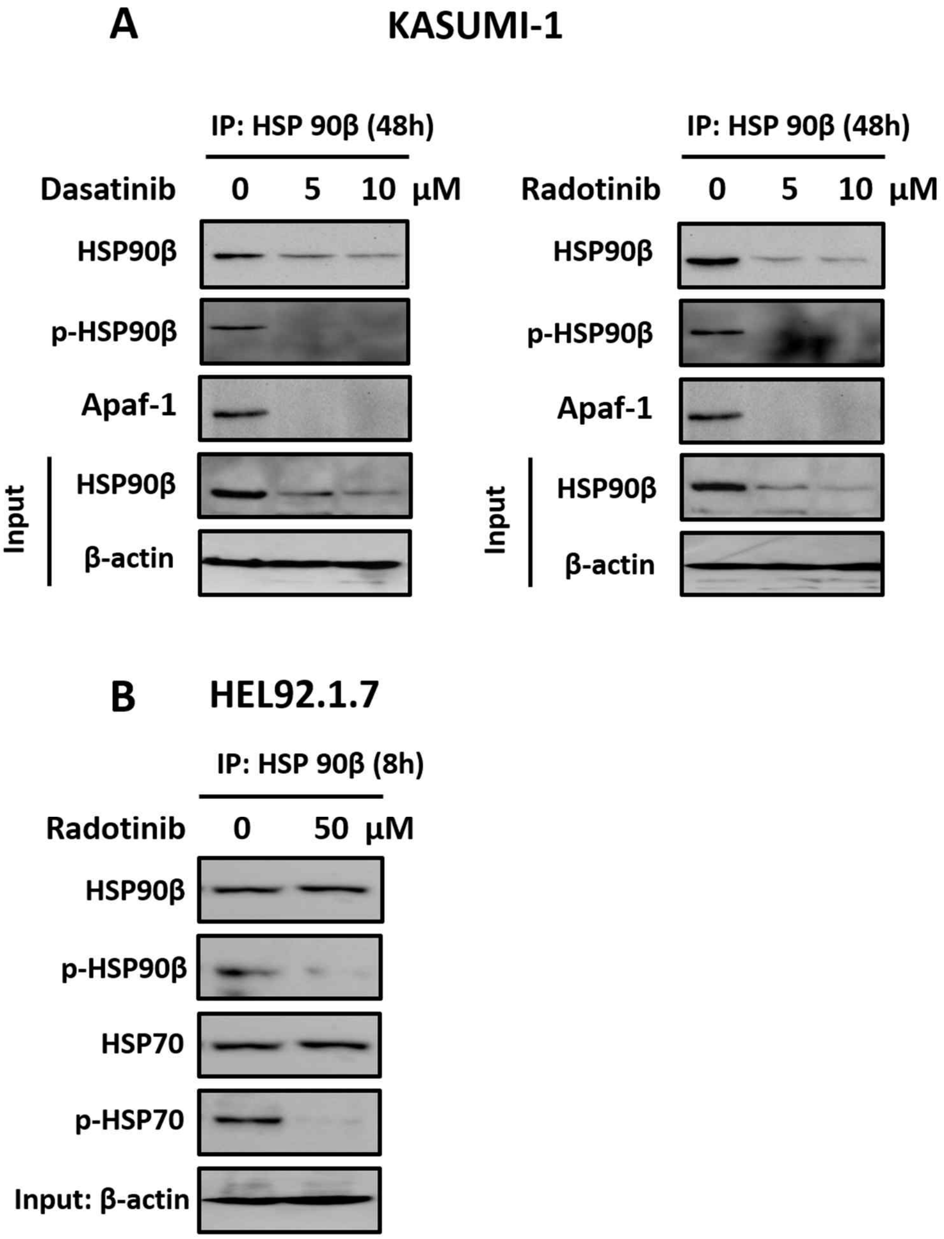

Supplementary Fig. 7. Dasatinib and radotinib inhibit HSP90β activity and expression in c-KIT-positive cells and regulate its binding with Apaf-1. (A) The HSP90β activity and expression by dasatinib or radotinib treatment at 48 h in KASUMI-1 cells. After 48 h, HSP90β was immunoprecipitated from KASUMI-1 samples treated as shown, and Western blotting shows the expression of phospho- and total HSP90β, and Apaf-1. (B) The expression of phospho- and total HSP70, and HSP90β at 8 h by radotinib treatment in HEL92.1.7. Each treatment was assayed in triplicate.

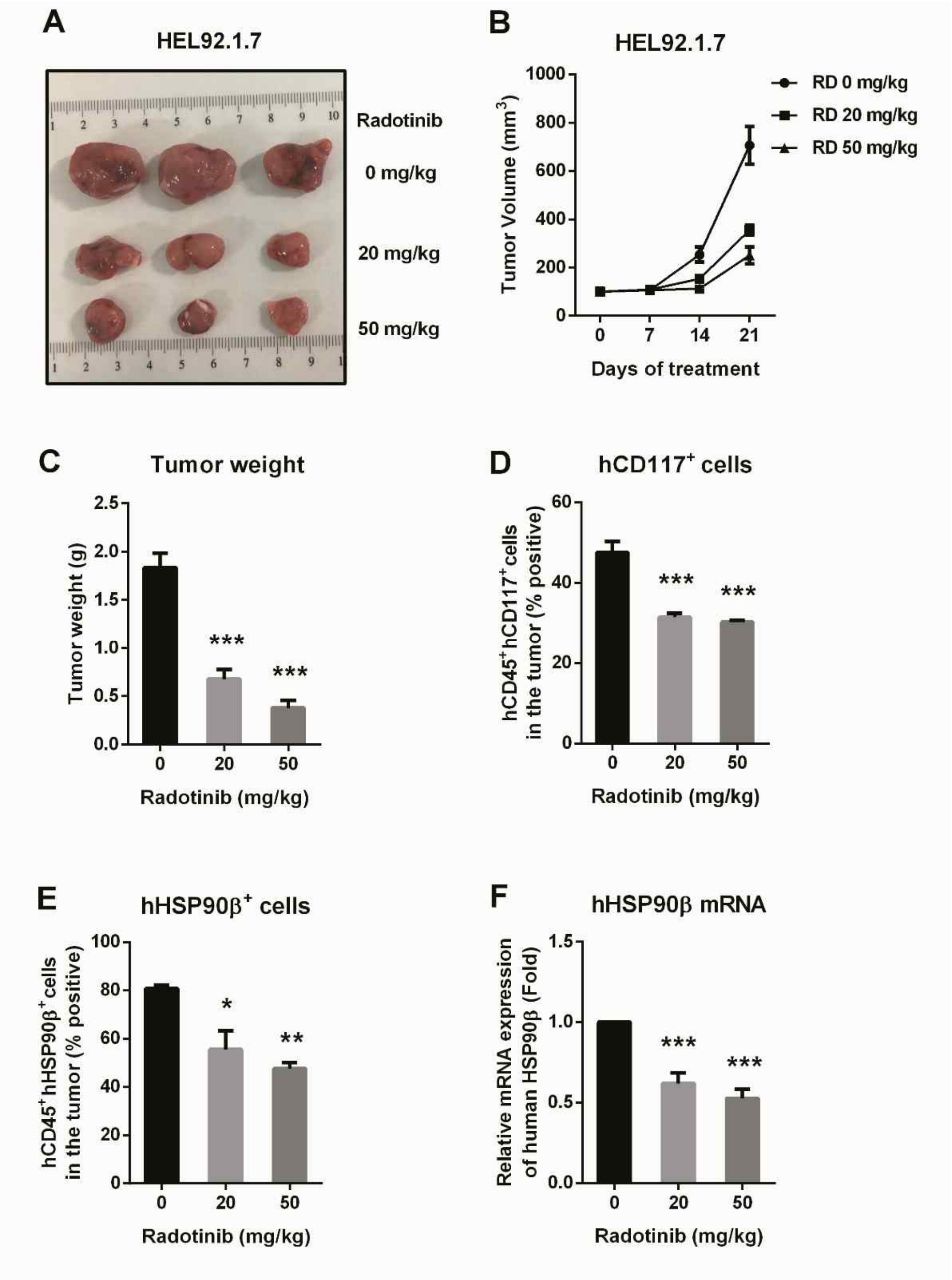

Supplementary Fig. 8. Radotinib inhibits tumor growth in the xenograft animal model using HEL92.1.7 cells (n = 4 for each group). (A) Representative photographs of tumors 24 days after radotinib treatment. When the tumors were ~ 150 mm^3 in size at ~ 7 days post-implantation, 0.2 ml radotinib (0, 20, 50 mg/kg body weight) was injected intraperitoneally 5 times per week, on different

days. (B) Tumor volume (mm^3). Tumor sizes were measured once a week using a digital caliper, and tumor volumes were calculated using the formula: (length × $width^2$) × 0.5. (C) Tumor weight (g). (D) The expression of human $CD45^+$ $c\text{-}KIT^+$ cells in the tumor. (E) The expression of human $CD45^+$ $HSP90^+$ cells in the tumor. (F) The expression of human HSP90β mRNA in the tumor. These data represent the means ± SEM. Significantly different from the control (*); *, $p < 0.05$; **, $p < 0.01$; ***, $p < 0.001$.

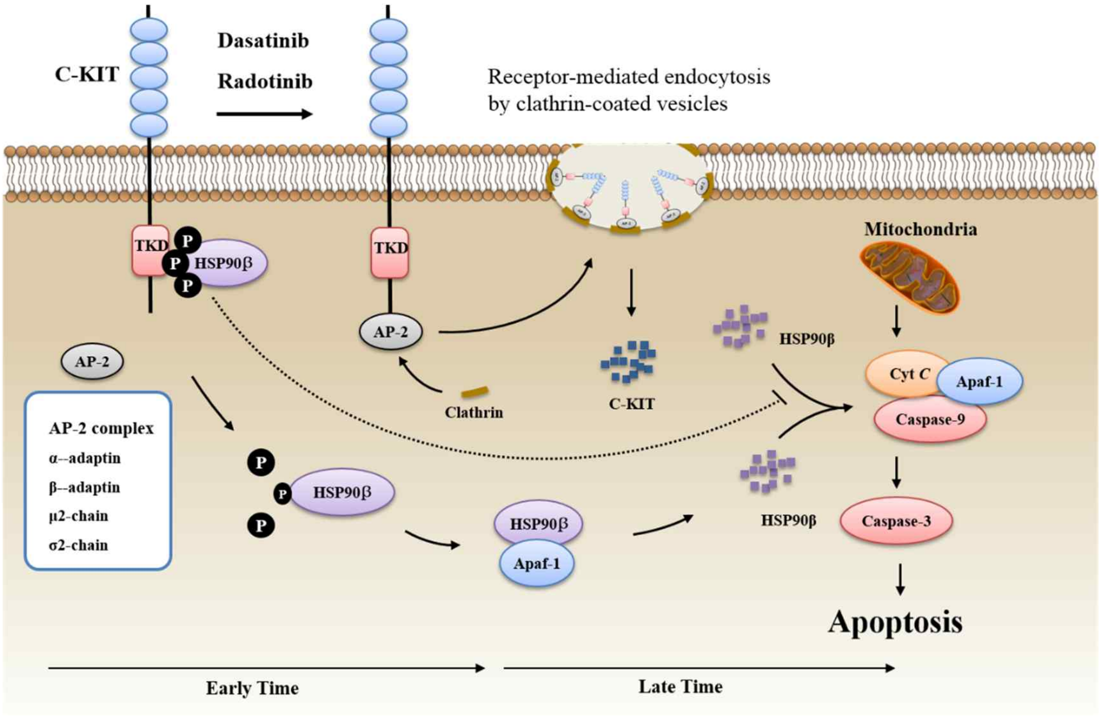

Supplementary Fig. 9. Targeting c-KIT by dasatinib and radotinib promotes acute myeloid leukemia cell death by suppression of HSP90β. In other words, c-KIT suppression/degradation by dasatinib and radotinib is essential for AML cell death via apoptotic pathway activation. At the same time, they regulate HSP90β activity and expression and its binding with other signaling molecules including c-KIT and Apaf-1. Eventually, dasatinib and radotinib remarkably induce AML cell death.
